# Dataset on the numbers and proportion of mortality attributable to hunting, trapping, and powerlines in wild and captive-bred migratory Asian houbara *Chlamydotis macqueenii*

**DOI:** 10.1016/j.dib.2018.10.154

**Published:** 2018-11-03

**Authors:** Robert J. Burnside, Nigel J. Collar, Paul M. Dolman

**Affiliations:** a School of Environmental Sciences, University of East Anglia, Norwich NR4 7TJ, UK; b BirdLife International, Pembroke Street, Cambridge CB2 3QZ, UK; c School of Biological Sciences, University of East Anglia, Norwich NR4 7TJ, UK

## Abstract

The data shown in this article are the number and proportion of deaths attributable to hunting/trapping, powerline collisions and natural causes in migratory Asian houbara *Chlamydotis macqueenii* originating from a breeding population in Uzbekistan. For wild adult and juvenile Asian houbara wintering in Central Asia during the period 2011–2018, 53.9% and 52.9%, respectively, of mortality was due to hunting/trapping, while in contrast most mortality in captive-bred released birds was natural with only 23.3% attributable to hunting/trapping. In winter, only one instance of powerline mortality was confirmed (6.0%). For wild adults, 23.0% of mortality during the summer was due to hunting/trapping, while 30.7% was due to powerline collisions. This data article is related to the publication “Captive breeding cannot sustain migratory Asian houbara *Chlamydotis macqueenii* without hunting controls” (Dolman et al., 2018) where further interpretation of the population-level consequences of these mortality causes can be found.

**Specifications table**

**Table:** 

Subject area	*Biology*
More specific subject area	*Conservation biology, ecology*
Type of data	*Table*
How data was acquired	*Animal telemetry with satellite tracking using Microwave Telemetry PTT-100 Argos/GPS solar-powered 30 g and 45 g transmitters.*
Data format	*Raw and analysed*
Experimental factors	*Three groups: captive-bred released houbara; wild adult houbara; wild juvenile houbara.*
Experimental features	*Annual cycle of Asian houbara migration between breeding and wintering areas.*
Data source location	*Uzbekistan, Turkmenistan, Iran, Afghanistan, Pakistan, Iraq*
Data accessibility	*Data are with this article.*
Related research article	P.M. Dolman, N.J. Collar, R.J. Burnside. Captive breeding cannot sustain migratory Asian houbara *Chlamydotis macqueenii* without hunting controls. Biol. Conserv., 2018, doi:10.1016/j.biocon.2018.10.001[1]

**Value of the data**

• Dataset on the causes of mortality for migrating birds on the Central Asian migratory flyway.
• Dataset provides a baseline estimate of the proportion of mortality due to hunting and trapping for Asian houbara for the period 2011–2018 and future studies can independently validate if implementation of regulated hunting and trapping mitigation leads to changes in mortality rates due to hunting/trapping.
• These data provide a baseline measure of proportion of mortality due to powerlines for Asian houbara in Uzbekistan and can be used to inform environmental impact assessments of powerline infrastructure throughout its range states.

## 1 Data

All raw data on mortality causes and percentages of mortalities attributable to hunting/trapping are shown in Table 1. The percentage of wild adult winter and wild juvenile first-winter mortalities attributable to hunting/trapping (excluding the Uzbekistan hunting concession, UHC) were similar (*t*_*28*_ = 0.049, *P* = 0.961), whereas for captive-bred released first-winter mortalities (excluding UHC), more birds died of natural causes, so the proportion attributed to hunting was significantly lower than for wild juvenile first-winter houbara (*t*_*45*_ = 2.148, *P* = 0. 0372). Of 13 adult summer mortalities, three were ‘sudden stop’ (of which two occurred in Uzbekistan and one in Afghanistan); six were considered ‘natural’ (of which three were confirmed in the field in Uzbekistan, and three were ‘static’, of which one was in Turkmenistan) and four were powerline mortalities in Uzbekistan (confirmed in the field).

**Table 1:** Number of mortalities observed in the period 2011–2018 and the causes of mortality for wild adult, wild juvenile and captive-bred released Asian houbara originating in Uzbekistan. See methods text for explanation of the attributed causes and their interpretation. Percentage of mortalities by hunting/trapping were calculated according to Eqs. (1), (2).

**Cohort**	**Period**	**Total number of mortalities during the period (excluding UHC**^a^**in brackets)**	**Attributed causes of mortality**					**Percentage of mortality by hunting/trapping**	
			**Known hunted/trapped (outside UHC**^**a**^**)**	**Sudden stop (likely hunted/trapped)**	**Known hunted/trapped (UHC**^**a**^**)**	**Static: natural death**	**Static: powerline**	**excluding UHC Eq.**(1)	**including UHC Eq.**(2)
Captive-bred	Winter	33 (30)	0	7	3	23	0	23.3% (SE 7.8%)	30.3% (SE 8.2%)
Wild juvenile	Winter	18 (17)	3	6	1	8	0	52.9% (SE 12.1%)	55.6% (SE 11.7%)
Wild adult	Winter	15 (13)	2	5	2	5	1	53.9% (SE 13.8%)	60.0% (SE 12.7%)
Wild adult	Summer	13	NA	3	NA	6	4	NA	23.0% (SE 11.7%)

a UHC: Licensed Uzbekistan hunting concession in Bukhara province of Uzbekistan.

## 2 Experimental design, materials, and methods

### 2.1 Tracking Asian houbara

Asian houbara originating in the Bukhara province, Uzbekistan, were tracked between autumn 2011 and summer 2018 using Microwave Telemetry PTT-100 solar-powered Argos/GPS satellite transmitters (hereafter PTT) attached to the birds using backpack harnesses held by Teflon ribbon. A total of 52 wild adults, 27 wild juveniles and 42 captive-bred released houbara were tracked over the winter periods, while a total of 58 wild adults were tracked during summer periods. Captive-bred birds were bred through artificial insemination and reared in captivity until their release in either the spring or the summer prior to the migration period. Wild juveniles and captive-bred released birds undertake their first migration and winter period as naïve migrants with no prior knowledge of migration routes or wintering sites and probably undertake these migrations alone [2]. Wild adults have already established their migration routes and wintering sites. The study area and population are within Bukhara, which is a licensed hunting concession (UHC). The aim of the associated research article [1] was to determine the mortality rates when no hunting is occurring within the UHC. Therefore, estimates below were calculated both with, and without, birds hunted in the UHC.

### 2.2 Classification of cause of mortality

PTT transmissions were checked every three days for signs of mortality interpreted from PTT location and engineering (voltage, temperature, activity sensor) data (following [3]). Whenever possible mortalities that occurred within Uzbekistan were examined in the field to assess the cause and confirm the engineering data interpretation methodology. This was done by carefully searching the location of the last GPS fix or when available by using UHF ground-track signals to locate the PTT. In this way, mortalities were classified into four groups:

• ‘Known hunted/trapped’:
  The following cases were all classified as known hunted/trapped: a) individuals hunted in the Bukhara concession (UHC) by the licensed hunt, and mortalities occurring in the wintering range where a hunter contacted us (via details printed on the transmitter); b) when transmissions showed the PTT had moved from houbara habitat to a human settlement (visible on Google Earth), from which static transmissions sometimes continued; or c) when the transmitter was transported via an airport or shipping port to a location in the Gulf.
• ‘Sudden stop’ (considered as likely hunted/trapped):
  Cases of sudden cessation of all transmissions (GPS, Argos and engineering data), when it was certain from PTT age, recent performance and engineering data that transmitter failure was highly unlikely [4], were interpreted as most likely attributable to hunting/trapping, as it is known that poachers or illegal hunters frequently destroy transmitters [4]. Although it is possible for a predator to damage a transmitter or leave a PTT in a position where it will not transmit, in our experience (based on recovery of transmitters following mortality within Bukhara) this is rare. In contrast to sudden stops, transmitter failure is typically preceded by progressive deterioration of the battery voltage with ever-increasing gaps in location and engineering data, and could readily be distinguished from mortality events.
• ‘Static: natural death’ (considered as non-anthropogenic; includes disease, starvation and/or predation):
  Following [3], continuous static transmissions from the same location for many days (or even months), with inactivity from the onboard sensors, were interpreted as predation (e.g. with the transmitter displaced from the carcass and static, or transmitting again after an interruption of transmission when initially predated and cached and then exhumed by a mammalian predator) and were classified as natural deaths. Similar instances were confirmed by field signs in Bukhara province, supporting interpretation of ‘natural mortality’ elsewhere in the flyway.
• ‘Static: powerline’:
  PTT data as for Static: natural death, with the addition that powerlines are visible on Google Earth satellite imagery and, on visiting a site, the remains of the bird and PTT are found under or close to the powerline. These interpretations were confirmed by Burnside et al. [5].

As independent support for these interpretations, we excluded ‘known hunt/trapped’ cases and then compared the relative frequency of ‘sudden stop’ to ‘static’, separately for three wintering range states with known year-round hunting and trapping activity (Iran, Afghanistan, Pakistan) and for Turkmenistan, a strongly regulated nation with tightly controlled borders from which we have no knowledge of houbara trapping trade. We *a priori* predicted that the frequency of ‘sudden stop’ relative to ‘static’ mortalities would be substantially lower in Turkmenistan. We found that, for Iran, Afghanistan and Pakistan, 18 (42.9%) of the 42 mortalities were ‘sudden stop’; in contrast for Turkmenistan only 2 (15.4%) of 13 mortalities were ‘sudden stop’ (one-tailed Fisher Exact Test, *P* = 0.0512), consistent with our hypothesis that the proportion of ‘sudden stop’ mortalities would be greater in wintering range states with more hunting/catching.

### 2.3 Estimating the proportionate contribution of hunting/poaching to mortality

Excluding all mortalities from licensed hunting within the UHC (that are not considered as part of the flyway mitigation scenario – see [1]), the proportion of winter mortality of satellite-tagged birds probably attributable to hunting was estimated as:

(1) $$\frac{({\mathrm{KnHT}}^{\mathrm{exUHC}}\;+\;\mathrm{Sudden})\;}{({\mathrm{KnHT}}^{\mathrm{exUHC}}\;+\;\mathrm{Sudden}\;+\;\mathrm{Static})}$$

where *KnHT*^*exUHC*^ were mortalities classified as ‘Known hunted/trapped’ outside the UHC, *Sudden* were ‘sudden stop’ and *Static* were classified as ‘static’ (as defined above). The overall proportion of winter mortality due to hunting, inclusive of the UHC, was estimated using the equation above, but including ‘Known hunted/trapped’ inside the UHC (*KnHT*^*UHC*^):

(2) $$\frac{({\mathrm{KnHT}}^{\mathrm{exUHC}}\;+{\;\mathrm{KnHT}}^{\mathrm{UHC}}\;+\;\mathrm{Sudden})\;}{({\mathrm{KnHT}}^{\mathrm{exUHC}}\;+{\;\mathrm{KnHT}}^{\mathrm{UHC}}\;+\;\mathrm{Sudden}\;+\;\mathrm{Static})}$$

Standard errors were estimated using a binomial generalized linear model with logit link [1]. Proportions were then converted to percentages.
