# Supplementary material for: Dataset on the numbers and proportion of mortality attributable to hunting, trapping, and powerlines in wild and captive-bred migratory Asian houbara Chlamydotis macqueenii
Source: Data Brief. 2018 Nov 3;21:1848–52. doi: 10.1016/j.dib.2018.10.154 (PMC6260302; doi:10.1016/j.dib.2018.10.154)
Supplement: Supplementary file 1 — Supplementary material [file mmc1.docx]

Conflict of Interest statement for DIB-D-18-02663

The Ahmed bin Zayed Charitable Foundation funded this work as part of a wider research collaboration of the Emirates Bird Breeding Centre of Uzbekistan, BirdLife International (BLI) and University of East Anglia (UEA), UK. BLI sub-contracted UEA to undertake independent and objective evaluation of strategies using fieldwork and satellite telemetry; all analyses were conducted independently by UEA, and other partners had no influence on magnitude or direction of findings. BirdLife co-authored the paper, EBBCC were informed of results and discussed interpretation. Research protocols have been approved by the UEA Animal Welfare and Ethical Review Body (AWERB).
